# Adsorption and Reaction of CO on (Pd–)Al_2_O_3_ and (Pd–)ZrO_2_: Vibrational Spectroscopy of Carbonate Formation

**DOI:** 10.1007/s11244-017-0852-7

**Published:** 2017-08-18

**Authors:** Karin Föttinger, Waltraud Emhofer, David Lennon, Günther Rupprechter

**Affiliations:** 1 Institute of Materials Chemistry, Technische Universität Wien, Getreidemarkt 9/BC/01, 1060 Vienna, Austria; 2 School of Chemistry, University of Glasgow, Joseph Black Building, University Avenue, Glasgow, G12 8QQ Scotland, UK

**Keywords:** Palladium-Zirconia, Alumina, Carbonates, IR spectroscopy, Chloride poisoning, Isotope labelling

## Abstract

γ-Alumina is widely used as an oxide support in catalysis, and palladium nanoparticles supported by alumina represent one of the most frequently used dispersed metals. The surface sites of the catalysts are often probed via FTIR spectroscopy upon CO adsorption, which may result in the formation of surface carbonate species. We have examined this process in detail utilizing FTIR to monitor carbonate formation on γ-alumina and zirconia upon exposure to isotopically labelled and unlabelled CO and CO_2_. The same was carried out for well-defined Pd nanoparticles supported on Al_2_O_3_ or ZrO_2_. A water gas shift reaction of CO with surface hydroxyls was detected, which requires surface defect sites and adjacent OH groups. Furthermore, we have studied the effect of Cl synthesis residues, leading to strongly reduced carbonate formation and changes in the OH region (isolated OH groups were partly replaced or were even absent). To corroborate this finding, samples were deliberately poisoned with Cl to an extent comparable to that of synthesis residues, as confirmed by Auger electron spectroscopy. For catalysts prepared from Cl-containing precursors a new CO band at 2164 cm^−1^ was observed in the carbonyl region, which was ascribed to Pd interacting with Cl. Finally, the FTIR measurements were complemented by quantification of the amount of carbonates formed via chemisorption, which provides a tool to determine the concentration of reactive defect sites on the alumina surface.

## Introduction

γ-Alumina is widely used as oxide support in catalysis. Due to its acidity, alumina is itself a catalyst, providing coordinatively unsaturated (*cus*) Al^3+^ sites acting as Lewis acid sites, as well as acidic surface OH groups. Frequently, monitoring the adsorption of molecules such as CO or CO_2_ by Fourier transform Infrared (FTIR) spectroscopy is utilized to characterize the acidic and basic sites of oxides (e.g. [1–4]).

In this field, pioneering work has been done by Helmut Knözinger and coworkers, and we refer to a number of in-depth reviews [5, 6]. The same group has also dealt with metal-support interactions, including sintering, redispersion and alloy formation [7]; topics that are still up to date [8–10]. Alumina supported palladium nanoparticles are among the most frequently used catalytic systems in industry, due to their exceptional properties for selective hydrogenation and oxidation. The adsorption of CO, H_2_ and other probe molecules/reactants on Pd–alumina has been extensively studied, providing a wealth of information on morphological properties of the metal nanoparticles (particle size, structure and surface roughness, electronic structure, etc (see e.g. [11–14]) and references therein.

The systems studied include single crystals, UHV-grown model nanoparticles and technical Pd catalysts on different support materials [12, 15–18]. Nevertheless, the interpretation of experimental results, *e.g*. those obtained by infrared spectroscopy or temperature-programmed methods, is still not unambiguous. For instance, the formation of carbonates has been assigned to CO dissociation/disproportionation on Pd (2 CO → CO_2_ + C) followed by reaction of the produced CO_2_ with the support [19, 20], despite a considerable body of literature (both experimental and theoretical) that rather excludes CO dissociation on Pd (for CO pressures up to 1 bar and temperatures up to 500 K) [21–24]. Alternative explanations that have been reported include the formation of carbonates via “water gas shift” reaction with hydroxyl groups of the support oxide (CO + OH → CO_2_ + H; followed by CO_2_ reaction with other OH groups or oxygen at the oxide surface) [19, 25–27], the presence of carbonates already before CO adsorption [28] and CO oxidation by O_ads_ captured on Pd [29]. Furthermore, the effect of Cl synthesis residues suppressing carbonate formation was explained by Cl suppressing CO dissociation [20].

**Scheme 1:**
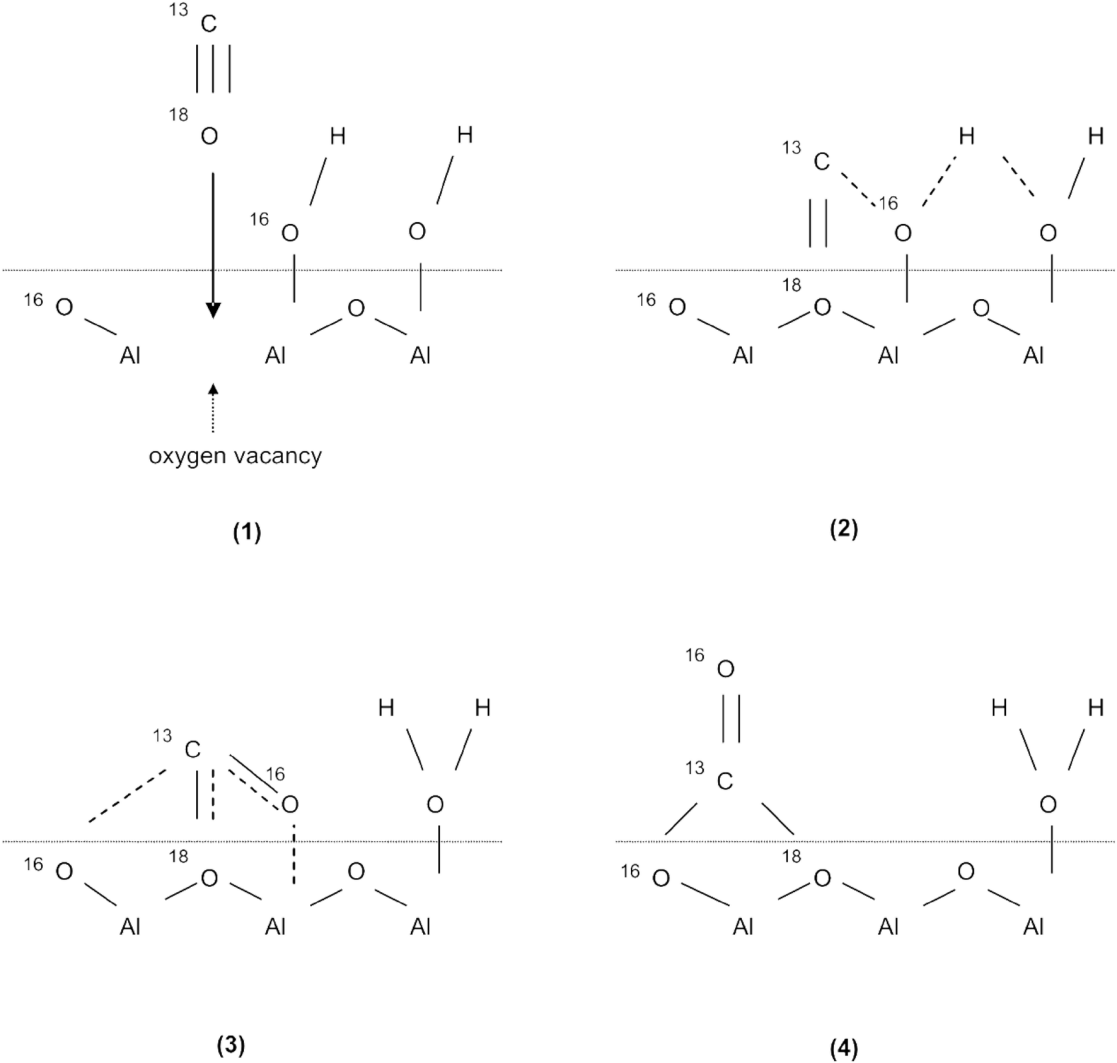
Mechanism of carbonate formation on Al_2_O_3_ ([26], reproduced by permission of the Royal Society of Chemistry)

These diverse considerations illustrate that there is a need to obtain an unambiguous answer on how carbonates are formed. A further interest in the formation of carbonates on metal oxide surfaces arises from the observation that they are involved in a series of reactions, especially related to CO oxidation and PROX [30], and synthesis gas production [31, 32]. For various reactions and for different oxides, carbonates have been suggested to be intermediates, but also to be spectators or even poisoning active sites. In a previous report [26] based on isotopic IR studies, we proposed a mechanism of carbonate formation (Scheme 1), which will serve as starting point for the current contribution. The suggested mechanism was confirmed employing comparative IR and XPS studies of UHV-grown alumina thin film model catalysts [27].

In brief, by utilizing isotopically labelled und unlabelled CO and CO_2_ the “water gas shift” mechanism of carbonate formation from CO + OH was identified to take place both on Pd–Al_2_O_3_ as well as on pure Al_2_O_3_. The proposed reaction scheme includes an “oxygen down” interaction of CO with a surface defect and further reaction with an adjacent hydroxyl group.

We have thus applied FTIR spectroscopy to follow room temperature carbonate formation upon catalyst exposure to CO and CO_2_. Two different oxides, γ-alumina and zirconia, with different acid/base properties were examined, and also served as support for well-defined Pd nanoparticles. This approach allowed distinction between CO dissociation and the water gas shift route. In a further step, we have studied the effect of Cl contaminants (synthesis residues) on carbonate formation. For this purpose, samples were deliberately Cl-poisoned. Finally, isotopically labelled CO and CO_2_ were employed to obtain mechanistic insights on carbonate formation. FTIR measurements were complemented by quantification of the amount of formed carbonates via volumetric adsorption.

## Experimental

### Catalysts Preparation

Commercial γ-alumina obtained from Sasol Germany GmbH (Puralox SBA 200, specific surface area 226 m^2^/g) was used in this study. Alumina supported Pd catalysts containing 2 and 5 wt% Pd were synthesized via incipient wetness impregnation using as precursors Pd(II)nitrate dihydrate (Fluka) and Pd(II)chloride (Fluka) in aqueous solution. All samples were stirred at room temperature for 1 h and dried at 373 K overnight. Calcination of the Pd/Al_2_O_3_ materials and of the pure Al_2_O_3_ were carried out at 773 K for 3 h in static air. The catalysts were reduced in situ (in the IR cell) in pure hydrogen at 573 K, immediately before a respective measurement.

For preparing Pd on zirconia catalysts zirconium hydroxide (MEL, ZXO 880/01) was calcined in static air at 773 K for 3 h (heating ramp 2°/min) to obtain monoclinic zirconia (specific surface area 82 m^2^/g). Three Pd–ZrO_2_ catalysts with a 5 wt% Pd loading were again prepared by incipient wetness impregnation using as precursors either an aqueous solution of Pd(II)nitrate dihydrate (Fluka), Pd(II)chloride (Fluka) dissolved in water, or Pd(II)acetate (Fluka) dissolved in toluene. The samples were calcined at 773 K for 3 h (heating ramp 2°/min) as described above.

Furthermore, the pure Al_2_O_3_ and ZrO_2_ supports were deliberately poisoned with chloride via impregnation with 1 N aqueous HCl solution, leading to approximately 2 wt% of Cl present at the surface, as determined by AES. Pd–ZrO_2_ prepared from a Cl-free (PdII)acetate precursor, was impregnated with HCl as well.

The following denomination will be used below for alumina supported catalysts: Pdx with x = Pd loading (in wt%), “nt” for the nitrate precursor, “cl” for chloride precursor, “ac” for acetate precursor, “/cl” for samples treated with Cl upon deliberate poisoning. The 5% Pd–zirconia catalysts were labeled as PdZnt, PdZac and PdZcl for the samples prepared from different precursors, and additionally “/cl” for the deliberately Cl-poisoned PdZac sample.

### Characterization

Specific surface areas were measured by N_2_ adsorption (BET method) using a Quantasorb apparatus equipped with a thermal conductivity detector. In order to find appropriate pre-treatment conditions temperature programmed reduction (TPR) (of unreduced catalysts) was carried out in a flow of 5 vol% H_2_ in He at a heating rate of 5 K/min (following a pretreatment in He flow at 573 K to remove water from the surface). The consumption of hydrogen and formation of water was followed by a mass spectrometer (Balzers QMS 200) and by a thermal conductivity detector.

The metal particle dispersion of the different Pd catalysts, reduced at 573 K in pure hydrogen, was characterized by combined (ex situ) high resolution TEM-EDX measurements (FEI Tecnai F20-FEG S-Twin TEM operated at 200 kV). The sample preparation was carried out by dipping holey carbon-coated gold grids into the dry powders. In addition, powder X-ray diffraction patterns were collected on a Philips X`Pert PRO (XP2) using CuK_α_ radiation, estimating crystallite size via the Rietveld method (program TOPAS R 2.1, Bruker, 2003).

To determine the metal dispersion by adsorption, hydrogen chemisorption was carried out in a volumetric apparatus after in situ reduction at 573 K and keeping this temperature in vacuum (approx. 10^−6^ mbar) for 1 h to desorb hydrogen and formed water. Two consecutive H_2_ isotherms were measured at 293 K with evacuation at room temperature in between in order to distinguish between surface-adsorbed and absorbed hydrogen. The difference of the first isotherm data (reversible + irreversible adsorption) and the repeated isotherm data (only reversible adsorption) was utilized to calculate the quantity of irreversibly adsorbed hydrogen. The mean Pd particle size was calculated based on the amount of chemisorbed (irreversibly adsorbed) hydrogen assuming spherical particles and a Pd:H ratio of 1.

Quantitative CO and CO_2_ adsorption measurements were performed in the same volumetric apparatus. To quantify the amount of CO adsorbed on the support, the amount of CO adsorbed on the metal particles was calculated assuming a CO:Pd ratio of 0.5.

Auger electron spectroscopy was applied to measure the concentration of chlorine residues originating from synthesis or deliberate poisoning. The spectra were recorded on a High Resolution Auger Electron Microscope VG Microlab 310 F equipped with a hemispherical analyzer. Powder samples were pressed onto pieces of Indium foil of about 1 cm^2^ size and glued to the sample holder.

### Transmission FT-IR Spectroscopy

Transmission FT-IR spectra were recorded on a Bruker IFS 28 spectrometer (MCT detector, resolution 4 cm^−1^). The catalysts were pressed to self-supporting wafers (amount of catalyst: approx. 10 mg), mounted on a ring-shaped furnace with a type K thermocouple and placed inside an IR cell equipped with CaF_2_ windows, which allowed exposure to different atmospheres up to 800 K. Additionally, the IR cell was equipped with liquid nitrogen cooling, which allowed measurements at low temperature (~100 K). Adsorption of probe molecules or reactants was carried out under static vacuum conditions with the gases introduced via a leak valve.

Prior to the adsorption studies, the samples were reduced in the IR cell at 573 K for 30 min, followed by treatment in vacuum at the same temperature for 1 h, and cooled to 293 K in vacuum (approx. 10^−6^ mbar). Typically, CO adsorption was carried out at 293 K, exposing 5 mbar CO until saturation was obtained, and followed by evacuation of gas phase CO at the same temperature. Adsorption of CO_2_ was performed in the same way. Apart from ^12^C^16^O and ^12^C^16^O_2_, the following CO and CO_2_ isotopes (Isotec) were used: ^13^C^18^O (min. 99 atom% ^13^C, 95 atom% ^18^O), ^13^C^18^O_2_ (min. 99 atom% ^13^C, 97 atom% ^18^O) and ^13^C^16^O_2_ (min. 99 atom% ^13^C). The double labelled molecules specifically have the benefit to allow differentiation from residual gas adsorption (which would contain ^12^CO_x_, but which did not occur).

## Results and Discussion

### Catalyst Characterization

TPR measurements (not shown) of calcined Pd–alumina catalysts displayed a maximum in hydrogen consumption at 400 K, indicating that Pd is fully reduced at about 400 K. After reduction at 300 K, FTIR spectra of adsorbed CO were identical to those after reduction at 573 K and indicated a reduced Pd surface (no bands characteristic of CO on electron-deficient Pd^δ+^ were found). However, at 300 K only the surface was reduced whereas Pd oxide species in the particle “bulk” required higher reduction temperatures according to XRD and TPR.

The specific surface area (BET) was similar (about 200 m^2^/g) for all alumina supported catalysts containing Pd, indicating that impregnation with Pd resulted only in a small decrease of the specific surface area of alumina (226 m^2^/g). The specific surface area was 202 m^2^/g for Pd1nt, 206 m^2^/g for Pd2nt and 193 m^2^/g for Pd5nt. For the zirconia-based samples the specific surface area obtained by the BET method resulted in the following values: PdZac 72 m^2^/g, PdZcl 86 m^2^/g, and PdZnt 67 m^2^/g. In comparison with the surface area of clean ZrO_2_ (82 m^2^/g) the samples prepared from acetate and chloride precursors showed a nearly unchanged surface area, whereas the surface area of the nitrate sample was somewhat smaller.

**Fig. 1:**
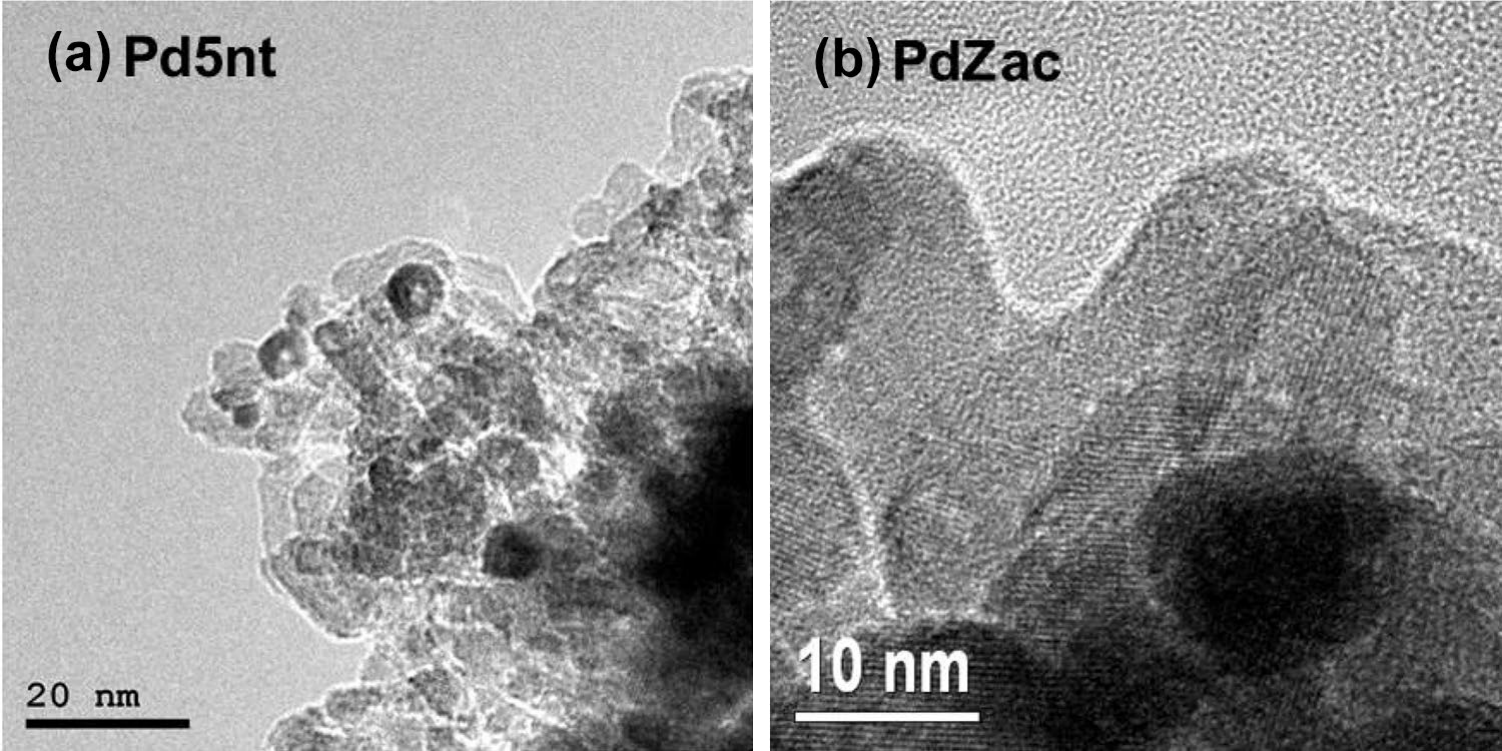
TEM images of Pd5nt (5 wt% Pd–Al_2_O_3_ prepared from Pd(II)nitrate precursor) and PdZac (5 wt% Pd–ZrO_2_ prepared from Pd(II)acetate precursor) after ex situ reduction

The size of oxide-supported Pd nanoparticles was determined by TEM and by quantitative H_2_ chemisorption. Figure 1a shows a TEM image of the Pd5nt catalyst showing well-facetted Pd particles. The mean diameter was about 5 nm. XRD patterns of Pd catalysts containing different metal loadings (2 and 5 wt%) were also acquired. However, estimation of the Pd crystallite size was only reasonable for 5 wt% Pd due to the low intensity reflexes observed for the lower metal loadings. Nevertheless, the mean crystallite size of 5.0 ± 0.5 nm agrees well with the TEM value. Volumetric H_2_ chemisorption resulted in mean Pd particle sizes of about 4.5 nm (25% dispersion) for Pd2nt and in similar values of 23% dispersion for Pd5nt. The catalyst prepared from the PdCl_2_-precursor exhibited smaller (average) Pd particles of about 3 nm, which corresponds to approximately 37% dispersion.

Pd–ZrO_2_ exhibited well-facetted Pd nanoparticles as well (Fig. 1b). Due to the similarity of the atomic weight of zirconium and palladium the contrast of the two elements in the images is similar, making it difficult to distinguish support and metal phase. By applying spatially resolved EDX analysis the identification of large Pd particles (around 10–15 nm) was confirmed, which most likely leads to an overestimation of the particle size obtained by TEM. H_2_ chemisorption, however, resulted in mean Pd particle sizes of 2.2 nm for PdZac, 3.2 nm for PdZcl and 2.9 nm for PdZnt. XRD of zirconia revealed mainly the monoclinic phase, with a minor amount of the tetragonal phase (10%). The mean size of the Pd crystallites was again calculated using the Rietveld method, resulting in 6.2 nm for PdZac, 7.2 nm for PdZcl and 4.8 nm for PdZnt. Thus, for the Pd–ZrO_2_ catalysts the determination of the mean Pd particle size did not lead to conclusive results, in contrast to Pd–Al_2_O_3_ materials.

AES was applied to measure the concentration of chloride residues after synthesis using Cl-containing metal precursors (Table 1). Due to the rather large errors for evaluating absolute concentrations the results of the quantitative Auger analysis have to be considered being rather an estimate, but they were nevertheless very useful during preparing the Cl-poisoned samples. For a comparison of the relative amount of Cl poisoning of different samples, the method is however clearly useful, and revealed between 1 and 2 wt% Cl content.

**Table 1:** Comparison of the Pd and Cl^−^ loadings in wt% of the Pd–ZrO_2_ catalysts determined by EDX, AES and obtained for the Pd phase from the Rietveld refinement of the XRD patterns

Sample	Synthesis	AES		EDX		XRD
	Pd	Cl^−^	Pd	Cl^−^	Pd	Pd
PdZnt	5	n.d	n.d	n.d	n.d	3.8
PdZac	5	0	6.3	0	1.8	3.7
PdZcl	5	1.9	7.6	2.1	0.8	2.9

*n.d*. not determined

### CO Adsorption on Pd–Al_2_O_3_ and Pd–ZrO_2_: Carbonyl Region

To assess the adsorption sites of the catalysts in detail we have performed vibrational spectroscopy of adsorbed CO. The main intention was to confirm that the catalyst were well-suited and well-characterized for the subsequent study of carbonate formation (Sect. 3.3. below), as well as to investigate the effect of Cl synthesis residues originating from different precursors in detail. The interpretation of the CO spectra was based on proceeding systematic studies of model [12, 33] and technical supported Pd catalysts [18].

**Fig. 2:**
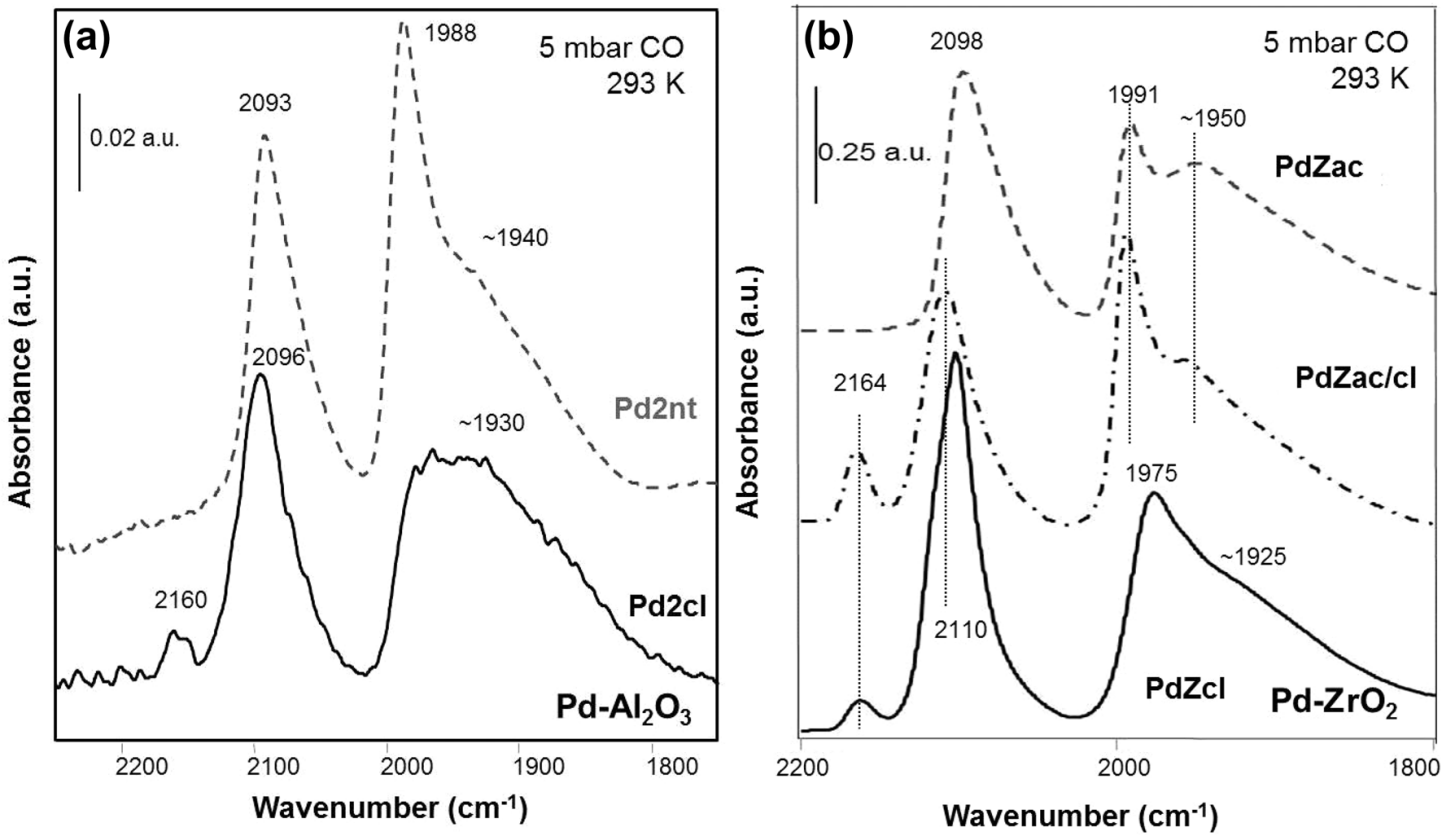
FTIR spectra of CO adsorption (5 mbar, 293 K) on **a** 2 wt% Pd–Al_2_O_3_ (prepared from nitrate and chloride precursors), and **b** 5 wt% Pd–ZrO_2_ catalysts (prepared from acetate precursor, acetate precursor followed by poisoning with Cl, chloride precursor) after reduction at 573 K: carbonyl region

Figure 2a compares CO spectra on the catalysts Pd2nt and Pd2cl, obtained at 293 K and 5 mbar CO pressure. Upon saturation of the Pd2nt catalyst with CO (exposure to 5 mbar for 15 min) the IR spectrum displayed bands at ~1940, 1988 and 2093 cm^− 1^, which were assigned to hollow or bridge bonded CO on (111) facets, to bridge bonded CO on particle edges and steps and (100) facets, and to on-top bonded CO, respectively. The spectrum is characteristic of ca. 0.5 ML CO coverage on well-facetted Pd nanoparticles, confirming that the Pdnt catalysts used for this study were clean and exhibited well-defined Pd nanoparticles. The origin of the IR bands and their assignment was based on experimental and theoretical work, as discussed in detail elsewhere (e.g. [11–14]).

As the most striking difference for Pd2cl catalyst, the adsorption of CO resulted in an additional band at 2160 cm^−1^ (Fig. 2a), which, to the best of our knowledge, has not been described before. Since this band was not present on pure alumina or the Cl-poisoned alumina, it can be attributed to CO adsorbed on electron deficient Pd, due to the interaction with Cl, whereas the idea of the band resulting from CO adsorption on *cus* Al^3+^ cations can be discarded. A comparable CO absorption band at about 2164 cm^− 1^ was also observed on the Pd–ZrO_2_ catalyst prepared from Pd(II)chloride, and also for the PdZac/cl catalyst that was poisoned by Cl treatment (Fig. 2b). This 2164 cm^− 1^ band was not present on PdZnt and PdZac though. Further, a peak at this frequency was not detected on pure and Cl^−^ treated zirconia. This strongly supports the suggestion that this band can be assigned to a CO adsorption site on Pd which is affected by Cl^−^ ions. This adsorption band proved to be very stable since it could not be removed by evacuation. In addition, the band of on-top adsorbed CO on Pd was shifted to slightly higher wavenumbers on PdZcl (from 2098 to 2110 cm^− 1^).

Supported Pd and Pt catalysts are often prepared from Cl precursors and it was frequently reported that Cl lowers the catalytic activity in oxidation reactions (e.g. methane combustion) (e.g.[34–36]). Cl residues were also shown to affect reaction selectivities, e.g. for hydrodechlorination on Pt–CeO_2_ catalysts [37]. Gracia et al. [36] investigated the effect of Cl poisoning on Pt–alumina and Pt–silica catalysts by EXAFS and IR of CO adsorption. On a Cl-containing catalyst that was prepared from a H_2_PtCl_6_ precursor, EXAFS detected Pt–Cl bonds in addition to Pt–Pt and Pt–O bands, and IR results showed a significantly decreased amount of adsorbed CO due to blocking of surface sites by Cl. It was reported that Cl was mobile on the Pt–alumina surface and was able to move back and forth between oxide and metal under reducing and oxidizing conditions.

### Carbonate Formation on Pd–Al_2_O_3_ and Al_2_O_3_

Reactive adsorption of CO has mainly been reported for oxides with basic character, such as MgO, CaO and ZrO_2_, leading to carbonate-type species with IR bands in the range 1700–1200 cm^−1^. Frequently, it was observed that carbonate formation requires elevated temperatures (above 473 K) [38]. The unambiguous identification of these species is not simple, because various different structures, but with similar IR signatures, can be formed, including free carbonate ions, mono- or bidentate carbonates, hydrogencarbonates, carbonites, carboxylate-type species (e.g. formates), etc. The assignment is further complicated because several different species may coexist. For alumina, the discussion on carbonates is controversial, since formation of carbonates seems strongly temperature dependent.

Despite their occurrence on (pure) oxide materials, the formation of carbonate-type species has repeatedly been assigned to CO dissociation on supported noble metal nanoparticles.

For Pd–MgO prepared from a chlorine-free Pd precursor, Bertarione et al. [20] attributed the appearance of carbonate bands upon room temperature CO adsorption to CO disproportionation on Pd (2CO → C + CO_2_). The CO_2_ produced would then react with the oxide support (with basic O_2_^−^ and OH^−^ ions) forming CO_3_^2−^ and HCO_3_^−^, whereas the carbon remains on Pd. For Pd–MgO and Pd–Al_2_O_3_ prepared from Pd–chloride, carbonate formation did not occur and this was explained by the poisoning of the metal with chlorine (preventing CO disproportionation) and by a reduced surface basicity of Al_2_O_3_ and Cl-poisoned MgO. A similar model, based on CO dissociation, was proposed in [39].

In contrast to this finding, studies of CO adsorption on Pd model surfaces typically reported non-dissociative CO adsorption [21, 22, 24, 40]. Combined near atmospheric pressure XPS and SFG investigations of CO interaction with smooth (annealed) and rough (ion-bombarded, “defect-rich”) Pd single crystals did not find indications for CO dissociation at mbar pressures of CO and 500 K [21, 22, 40]. At higher temperature Matolin et al. [41] reported defect-induced dissociation of CO on Pd (onset temperature of dissociation: approx. 410 K) whereas on annealed Pd only molecular adsorption occurred.

There are also reports of CO dissociation on Pd nanoparticles above 400 K (Rainer et al. [42], and Doering et al. [43]) suggesting that CO dissociation may occur at low coordinated defect sites where CO is stronger bonded and the C–O bond hence weakened. It seems that there is a strong influence of particle size, metal-support interaction, pressure and temperature, and defect sites, which may explain the varying results reported in the literature. One should also take into consideration that experiments using extended CO exposures at high temperature may be affected by undetected impurities (e.g. hydrocarbons) that could act as carbon source. Furthermore, Fe– and/or Ni–carbonyl impurities in the CO gas may eventually deposit Fe and/or Ni, that dissociate CO [12].

One should note that DFT [44] suggests a quite high activation barrier for CO dissociation on Pd. Liu et al. [45] reported that the CO dissociation barrier is strongly reduced on stepped and kinked Pd surfaces but this would still require temperatures around 500 K.

In any case, CO exposure around 300 K should not lead to CO dissociation. However, carbonate formation still occurs on our samples, i.e. there must be a source of CO_2_. As mentioned, explanations proposed in literature [19, 29] include CO disproportionation on the metal, water gas shift-reaction with hydroxyl groups of the oxide or that carbonates were already present before CO adsorption [28]. Also, reaction of CO with oxygen traces in the feed was proposed [29].

In a previously published study [26], we have investigated the mechanism of carbonate formation on a 2 wt% Pd–Al_2_O_3_ catalyst via adsorption of isotopically-labelled CO and CO_2_ and proposed a formation pathway consisting of reaction of CO with hydroxyl groups in a water–gas shift-like reaction, which will be elaborated in more detail in the following.

#### Reactive Adsorption of CO and CO_2_ on Pd–Al_2_O_3_ and Al_2_O_3_

CO adsorption on Pd–Al_2_O_3_ leads—apart from the molecular CO species on Pd—to additional bands in the range 1700–1200 cm^−1^, originating from carbonate and bicarbonate species (in the following the various species and structures including carbonates and bicarbonates will be termed “carbonate species”).

**Fig. 3:**
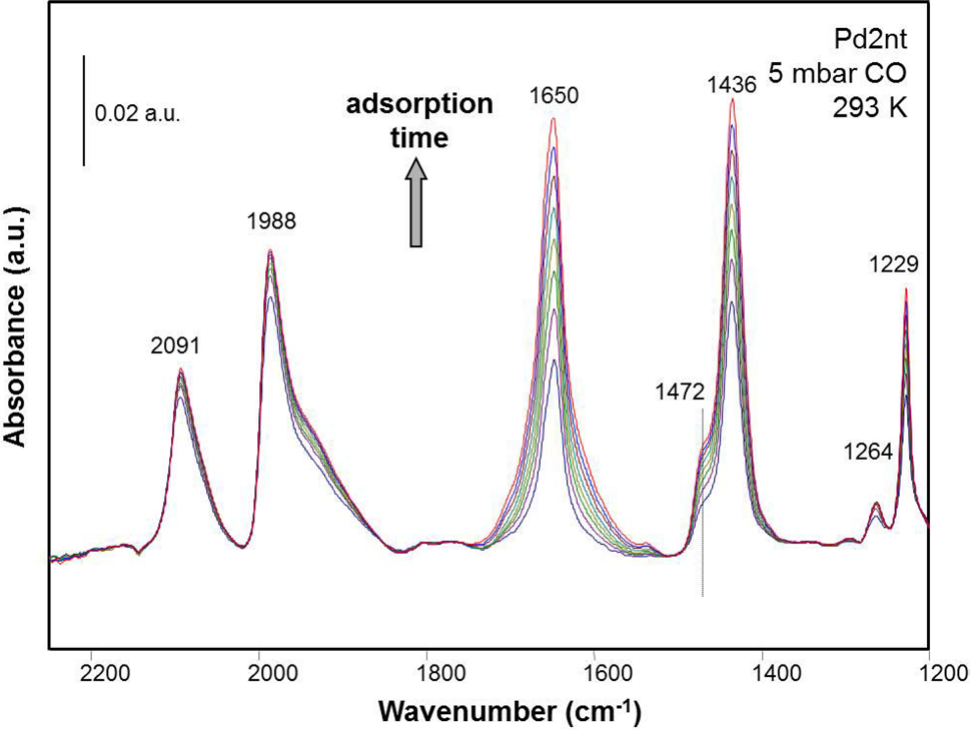
FTIR spectra of CO adsorption (5 mbar, 293 K) on Pd2nt (2 wt% Pd–Al_2_O_3_ prepared from Pd(II)nitrate precursor) with time on stream (TOS). Spectra of the carbonate region were recorded every 3 min. The first spectrum was recorded after 1 min exposure to CO

On Pd2nt carbonate bands were observed at these positions (Fig. 3): main peaks at 1650, 1436 and 1229 cm^−1^, a shoulder at 1472 and a weak peak at 1264 cm^−1^. The same band frequencies were found for the pure oxide support, as well as for CO_2_ adsorption on Pd–Al_2_O_3_ and Al_2_O_3_ [26]. Contamination of CO with CO_2_ or O_2_ can be excluded. The carbonate species grow with adsorption time in CO atmosphere, as is shown in Fig. 3.

The assignment of the IR peaks is not straightforward, because adsorbed carbonates can exist in different structures. Free carbonate ions on oxide surface give rise to a band at 1440 cm^−1^ (ν_as_CO_3_^2−^). Monodentate species are characterized by bands at 1530–1470 cm^−1^ (ν_as_COO^−^) and 1370–1300 cm^−1^ (ν_sy_COO^−^). Bidentate carbonates can exist in two different structures: chelating bidentate with bands at 1620–1530 cm^−1^ (νC=O) and 1270–1250 cm^−1^ (ν_as_COO) and bridging bidentate exhibiting vibrational bands at 1670–1650 cm^−1^ (νC=O) and 1270–1220 cm^−1^ (ν_as_COO). Frequently, bicarbonate species are observed giving rise to bands at 1625–1600 cm^−1^ (ν_as_COO), 1440–1415 cm^−1^ (ν_sy_COO) and 1250–1180 cm^−1^ (δCOH). In addition to carbonate species, also carboxylate-type structures can arise, such as formates, which lead to bands at 1630–1560 and 1420–1350 cm^−1^ (asymmetric and symmetric stretching vibrations of COO^−^, respectively) [46]. Upon formation of formate structures additional bands emerge due to C-H stretching vibrations. Formation of formates usually occurs at higher temperatures (>373 K) upon interaction of CO with hydroxyl groups of the oxide. However, formation of formate compounds can be excluded in our measurements, because no bands in the CH stretching region were observed.

In ref [38]. bands of bicarbonate and carbonate species evolved during heating in CO to 473 K: bands at 1659, 1648, 1438, 1230; further heating to 673 K: new bands at 1597, 1396, 1380; the authors assigned the first set of bands to hydrogencarbonate species, and the second to formate species. Similar assignments of the vibrational frequencies of reactive CO adsorption were reported in [47] and by Hadjiivanov et al. [3]. Furthermore, Hadjiivanov attributed bands at 1495–1478 cm^−1^ to carbonites (ν_as_CO_2_), and hydrogencarbonates were reported to appear at 1625–1600 cm^−1^ (ν_as_COO), 1440–1415 cm^−1^ (ν_sy_COO) and 1250–1180 cm^−1^ (δCOH).

Considering theses assignments, we can safely state that upon CO or CO_2_ adsorption on our catalysts mainly bicarbonates and/or bidentate carbonates were produced. Most likely, bicarbonates are the predominant species, which are formed during CO adsorption on the hydroxylated alumina surface.

These results directly indicate that carbonate formation cannot be (exclusively) attributed to CO dissociation on Pd. Assuming that CO does not dissociate on alumina, CO_2_ formation seems to occur via the water gas shift reaction (CO + OH → CO_2_ + H) followed by subsequent carbonate formation.

This assumption is further supported by changes observed in the OH stretching region occurring upon CO adsorption, these changes were discussed in our previous work [26]. Sharp OH bands were partially reduced upon CO exposure and a broad band at lower frequency appeared, whereas upon CO_2_ adsorption the sharp OH bands remained and no broad OH band was produced.

The negative OH band (due to consumption) appearing in difference spectra (cf. Fig. 1 in ref. [26]) at approximately 3770–3775 cm^− 1^ is typically attributed to the most reactive OH groups on the γ-alumina surface [2]. Morterra et al. ([2] and references therein) have attributed the OH band at 3775 cm^− 1^ to AI^IV^–OH groups present at locations of the surface related to crystallographic defects. These are expected to be quite frequent in porous systems with high surface area and poor crystallinity. Knözinger [48] proposed a very detailed and complete model for describing the OH groups present on transition aluminas and their vibrational frequencies based on the net electrical charge at the OH group which depends on the coordination number of the OH group and the Al cation. According to this model the higher frequency bands possess the highest basicity.

**Fig. 4:**
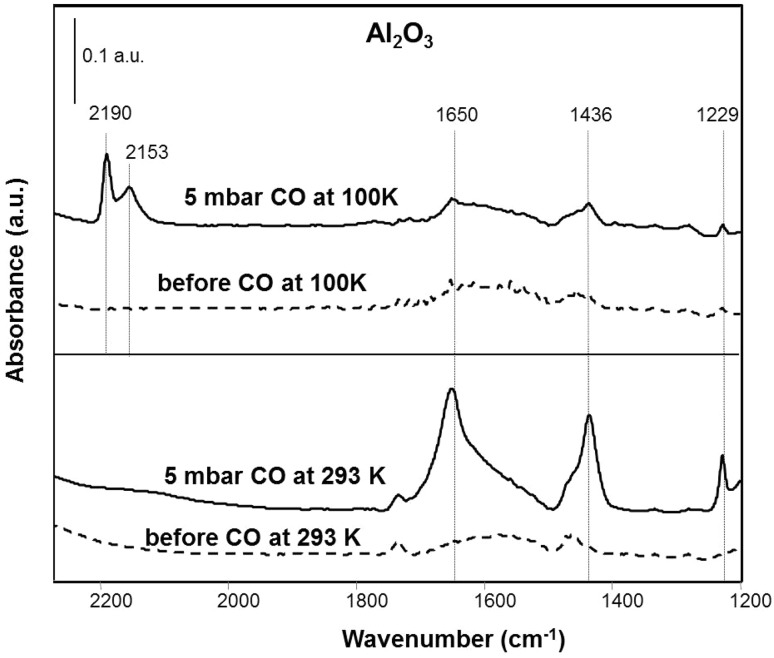
FTIR spectra of carbonate formation upon CO exposure (5 mbar) to Al_2_O_3_ at 100 K versus 293 K

According to temperature-dependent studies, carbonate formation seems to be an activated process that occurs slowly around room temperature on the pure oxide. This is illustrated in Fig. 4, comparing CO exposure at 100 K and at 293 K. The peaks at 2190 and 2153 cm^− 1^ at 100 K are due to CO adsorption on cus Al^3+^ sites and interaction with OH groups occurring at low temperatures [2].

However, in the presence of Pd, carbonate formation proceeds fast even at 100 K. In light of the weak interaction of CO with the alumina support and the much stronger interaction of CO with the metal, the faster formation of carbonate-type species points to a spill-over of adsorbed CO from Pd to the support. CO adsorbed on Pd may react with OH groups at the metal-support interface forming CO_2_, which would then react with the support. In fact, it is known that metals can enhance the activity for the water-gas-shift reaction of oxides, e.g. for Pt/ceria as compared to ceria alone [49].

**Fig. 5:**
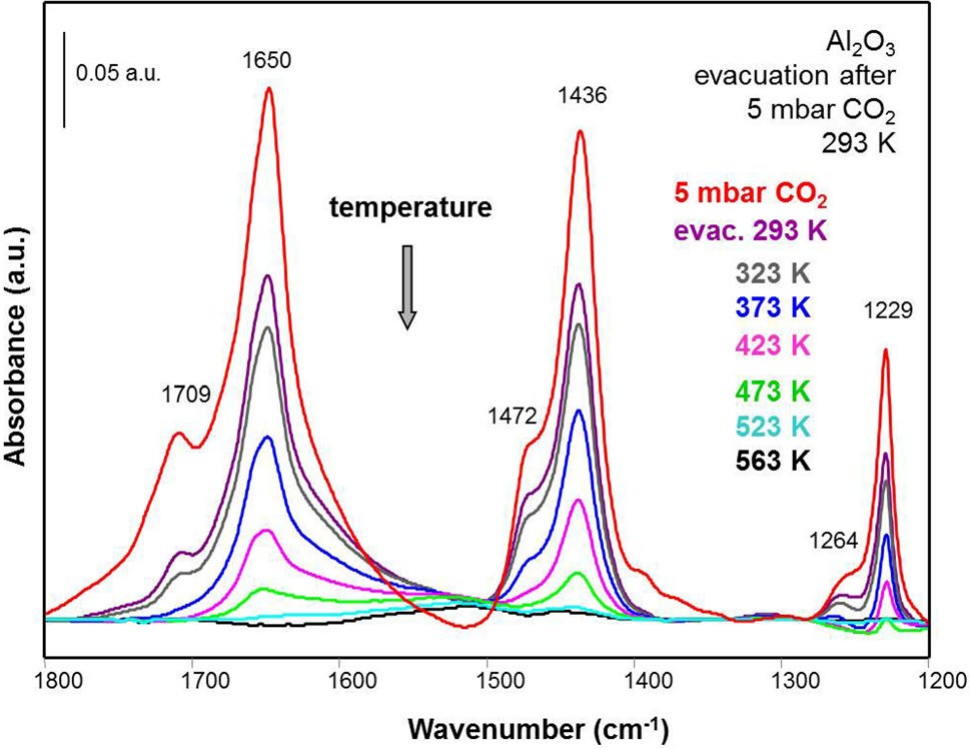
Thermal stability of the carbonates: adsorption of CO_2_ (5 mbar, 293 K) on Al_2_O_3_ followed by evacuation at 10^− 6^ mbar and 293 K for 15 min, and heating in vacuum with a rate of 2 K/min. Spectra were recorded every 10 K

To determine the thermal stability of the carbonate bands, temperature programmed desorption (TPD) was performed, using IR spectroscopy to monitor the various bands (heating rate 2 K/min at 10^−6^ mbar, IR spectra collected every 10 K; Fig. 5). The carbonate species continuously decreased in intensity and vanished at 523 K. The simultaneous intensity decrease of all bands with increasing temperature indicates the presence of carbonate species of the same type and/or the same thermal stability, *i.e*. bicarbonates and bidentate carbonates.

#### Influence of Cl Contaminants

**Fig. 6:**
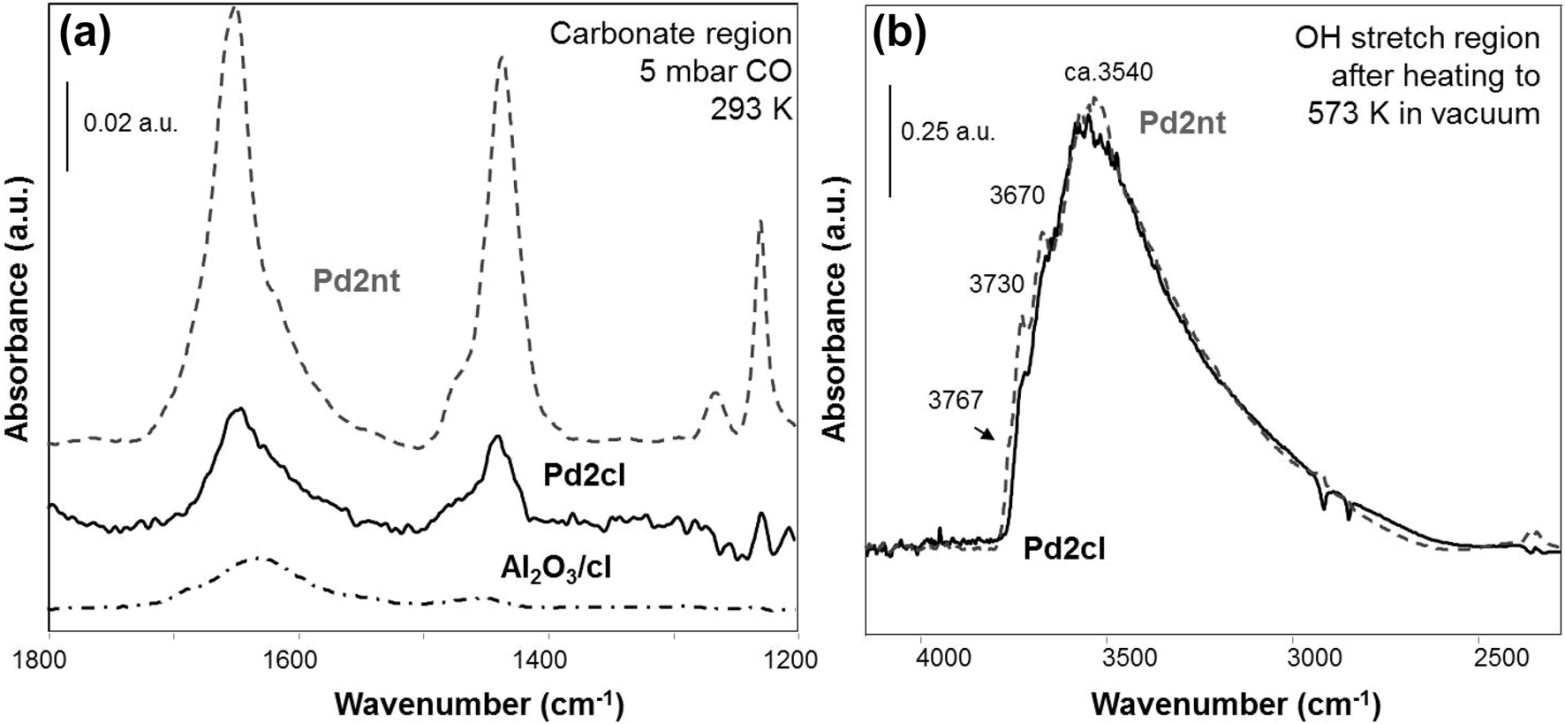
Effect of Cl contaminations: **a** Adsorption of 5 mbar CO at 293 K on Pd2nt, Pd2cl and Al_2_O_3_/cl. **b** OH stretching vibration region of Pd2nt and Pd2cl recorded at 293 K after activation in vacuum at 573 K. Pd2nt and Pd2cl correspond to 2 wt% Pd–Al_2_O_3_ prepared from Pd(II)nitrate and chloride precursors, respectively

To address the effect of synthesis residues (contaminants), CO adsorption experiments were carried out on catalysts prepared from Pd(II)chloride. The intensity of the carbonate bands was significantly reduced (see Fig. 6a), when compared to Pd2nt, in agreement with the results of Bertarione et al. [20]. Upon CO adsorption on Cl-poisoned alumina no carbonates were produced at 300 K (see Fig. 6a). Upon CO_2_ adsorption (not shown) on Pd2cl and Al_2_O_3_ deliberately poisoned with Cl reduced carbonate formation was observed.

These results suggest that the surface hydroxyl groups were strongly affected by contamination with chloride. A possible explanation is that support OH groups were substituted by Cl, which would reduce or inhibit the water gas shift reaction. The hindrance upon CO_2_ adsorption may indicate that OH groups are required to form bicarbonates or an effect of the modified acidity on carbonate formation. Indeed, the OH stretch region of the Cl-containing materials show somewhat reduced intensity of (high-frequency terminal) OH species (Fig. 6b). Note, however, that for samples without Cl residues carbonate formation from CO was accompanied by significant consumption of terminal OH groups [26]. Also Digne et al. [50] showed in a DFT simulation that Cl substituted preferably terminal OH groups (due to steric constraints and the O–H^…^O interaction stronger than O-H^…^Cl). This supports our explanation.

In summary, there is no need to invoke a Cl-poisoning of CO *dissociation* on Pd to explain the reduced carbonate formation. Rather, the effect of chlorine is attributed to a modification of surface OH groups and a hindrance of water gas shift and formation of CO_2_.

Concerning the effect of Cl^−^ on alumina it is known that chlorination leads to a strengthening of Lewis acidity of the surface. The adsorption of HCl was found to occur dissociatively via two different pathways, either by exchange with surface hydroxyl groups or by adsorption on aluminium–oxygen-pairs with formation of new hydroxyl groups adjacent to Al–Cl [51, 52].

#### Quantitative Determination of Carbonates

Quantitative chemisorption studies of CO were performed at 293 K in a volumetric system in order to estimate the amount of carbonates formed. Table 2 shows the values obtained from collecting isotherms at 293 K on the pure support and on the Pd containing catalyst Pd2nt.

**Table 2:** Quantification of carbonates formed on 2 wt% Pd–Al_2_O_3_ and Al_2_O_3_, based on CO or CO_2_ chemisorption isotherms at 293 K acquired in a volumetric system

Al_2_O_3_	Amount of adsorbed CO	2.20 × 10^− 8^ mol m^− 2^
	Amount of adsorbed CO_2_	8.52 × 10^− 7^ mol m^− 2^
Pd2nt	Total amount of adsorbed CO	1.18 × 10^− 7^ mol m^− 2^
	–CO adsorbed theoretically on Pd^a^	9.40 × 10^− 8^ mol m^− 2^
	=CO on oxide (carbonates)	2.38 × 10^− 8^ mol m^− 2^
	Amount of adsorbed CO_2_	8.55 × 10^− 7^ mol m^− 2^
Pd2cl	CO adsorption on support	Negligible
Al_2_O_3_/cl	CO adsorption	Negligible

^a^Assuming CO:Pd = 0.5; amount of Pd_surf_ obtained from H_2_ chemisorption

The number of Pd surface atoms was determined by hydrogen chemisorption and was also calculated from the mean particle size of 4.5 nm determined via TEM. Subtracting the amount of CO adsorbed on the Pd particles (for a CO coverage of 0.5 ML) indicates that about 20% of the overall amount of adsorbed CO reacts on the support and produces carbonates under our conditions. This corresponds well to the number of CO molecules adsorbed on pure Al_2_O_3_ (see Table 2).

Adsorption of CO_2_ leads to a higher uptake, which agrees with the observation of higher intensity of carbonate bands upon exposure to CO_2_. Furthermore, the same amount was adsorbed with and without Pd.

As a consequence, reactive adsorption of CO on alumina could be used as a selective probe for surface defects. On our alumina sample approximately 1.4.× 10^12^ carbonate molecules were formed on 1 cm^2^ surface area or 1.4 × 10^−2^ on 1 nm^2^ after pre-treatment at 573 K.

Additionally, one has to take into account that there is a possible impact on dispersion measurements via quantitative CO chemisorption. For example, in the case of Pd particles with a mean size of 4.5 nm (dispersion 25%), the particle size (dispersion) determined by CO chemisorption amounts 3.6 nm (31%), when carbonate formation on the support is ignored.

In agreement with FTIR spectroscopy, a clear effect of Cl contamination on the CO and CO_2_ uptake was observed. On catalyst prepared from Cl precursor and thus containing Cl residues the amount of CO adsorption was negligible (Table 2). The same holds for the deliberately Cl-poisoned alumina sample.

### Carbonate Formation on (Pd–)ZrO_2_

**Fig. 7:**
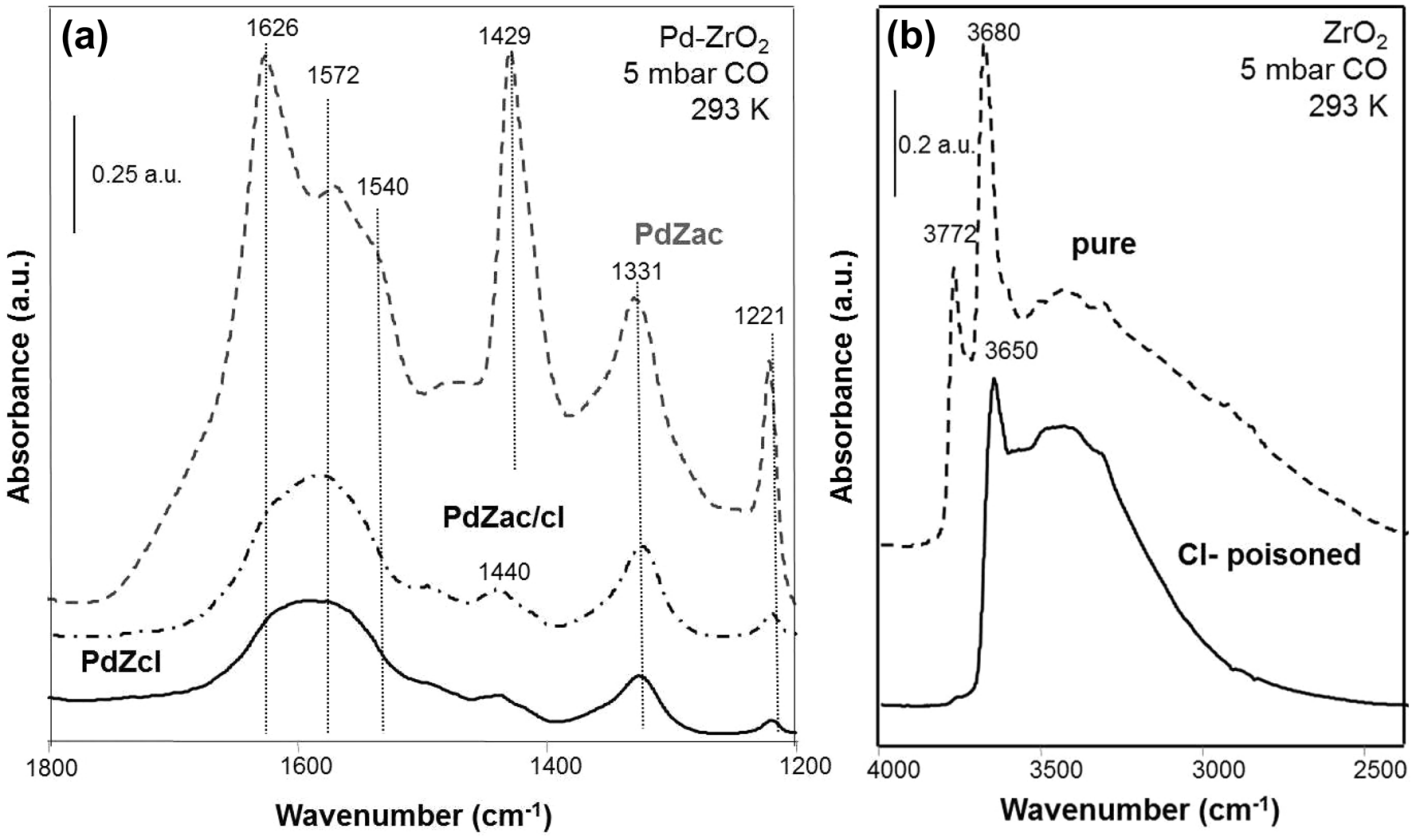
**a** CO adsorption (5 mbar, 293 K) on 5 wt% Pd–ZrO_2_ catalysts (PdZac, PdZac/cl, PdZcl) demonstrating the Cl poisoning effect. **b** OH stretching vibration region of pure and Cl-poisoned ZrO_2_ after activation in vacuum at 573 K

For comparison, carbonate formation was also investigated on a less acidic oxide, ZrO_2_, and the corresponding Pd-loaded samples. In general, a behavior very similar to that of alumina-based samples was observed. Carbonate bands were detected at the same frequencies for pure zirconia and samples loaded with Pd: at 1626, 1572, 1540, 1429, 1331 and 1221 cm^−1^ (Fig. 7a). A variety of different carbonate species was present on the zirconia surface. Assignment of the bands was based on literature (e.g. [53–56]). The vibrational bands at 1626, 1429 and 1221 cm^−1^ were attributed to surface bicarbonate species, while bands at 1572 or 1540 and 1331 cm^− 1^ can be assigned to bidentate carbonates (two slightly different species were reported as discussed in detail e.g. by Bachilla-Baeza [53]).

As on alumina-supported samples, the effect of the Cl-ions on the zirconia-based systems was to induce much weaker intensities in the carbonate range. This was observed for all Cl-containing materials, including deliberately poisoned ZrO_2_/cl, PdZac/cl and the catalyst prepared from the Pd(II)chloride precursor PdZcl (Fig. 7a). When closely inspecting the OH stretch region, pronounced changes were visible for the Cl-poisoned samples, indicating a partial substitution of OH groups by Cl, similar as for alumina (Fig. 7b). The band of isolated OH groups at 3772 cm^− 1^ disappeared nearly completely, whereas the intensity of the hydroxyl stretch vibration band at 3680 cm^− 1^ decreased considerably in intensity and was shifted to lower wavenumbers (from 3680 to 3650 cm^− 1^). Overall, very pronounced effects of Cl contaminants on the OH region of the ZrO_2_ samples were observed, which can again explain the strong decrease in surface carbonate species formed from CO and CO_2_ (the latter not shown).

In summary, these results already indicate that the mechanism of carbonate formation is basically the same on zirconia and alumina. To confirm this assumption, we have performed a mechanistic investigation using labelled compounds, as described in the following.

#### Reactive Adsorption of ^13^C^18^O and ^13^C^18^O_2_

**Fig. 8:**
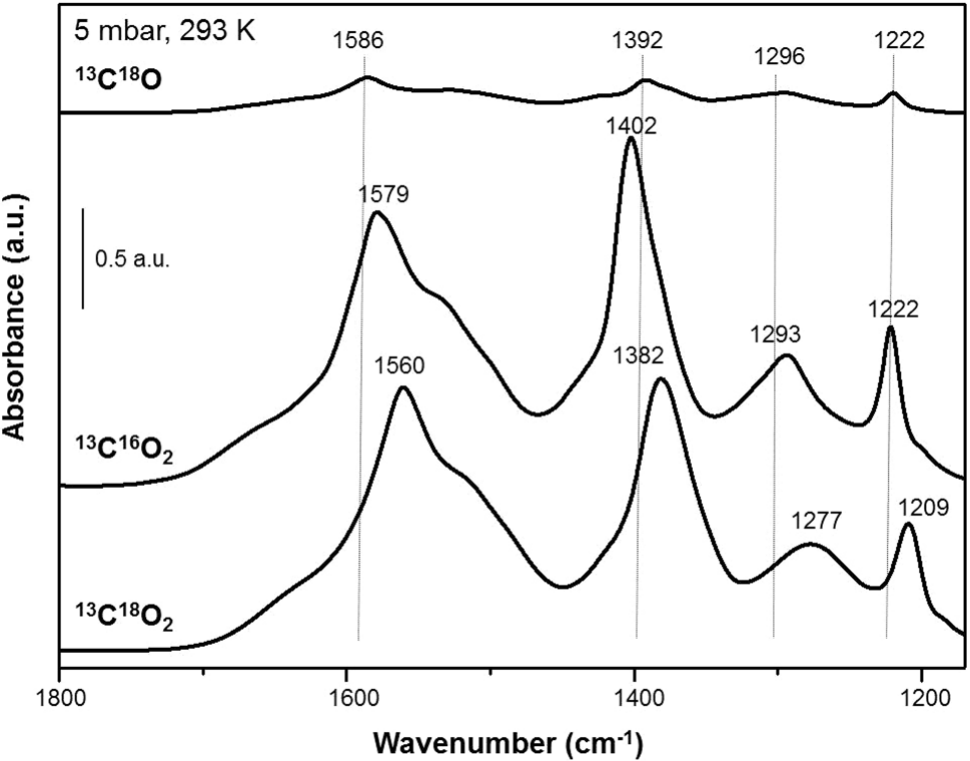
Adsorption of labelled molecules on ZrO_2_ (5 mbar, 293 K): ^13^C^18^O, ^13^C^16^O_2_ and ^13^C^18^O_2_

Our previous adsorption studies of labelled compounds on Pd–Al_2_O_3_ and Al_2_O_3_ [26] had clearly shown that carbonate formation occurs via CO reaction with surface hydroxyl groups, even at 300 K and in the absence of Pd. In order to exclude the CO dissociation pathway in the presence of Pd we utilized the adsorption of isotopically labelled ^13^C^18^O [26]. This experiment allowed us to unambiguosly distinguish between CO_2_ formation via CO disproportionation (2 ^13^C^18^O → ^13^C^18^O_2_ + ^13^C) and via water gas shift reaction (^13^C^18^O + ^16^OH_supp_. → ^13^C^18^O^16^O + ½ H_2_). Identification of the observed frequencies of the formed carbonates was achieved by performing adsorption experiments with ^13^C^18^O_2_ and ^13^C^16^O_2_. Similar experiments were also carried out for ZrO_2_ in an effort to find out whether the same surface reactions occur (Fig. 8). The vibration frequencies are compared to the alumina samples in Table 3.

**Table 3:** Positions of the main carbonate bands observed after adsorption of ^12^C^16^O, ^13^C^18^O, ^12^C^16^O_2_, ^13^C^16^O_2_ and ^13^C^18^O_2_ at 293 K (in all cases in a background pressure of 5 mbar) on Pd2nt (2 wt% Pd–Al_2_O_3_ prepared from Pd(II)nitrate precursor), Al_2_O_3_ and ZrO_2_

Material	Exposure of	Carbonate band positions (cm^− 1^)
Pd2nt, Al_2_O_3_	^12^C^16^O	1650, 1436, 1229
	^13^C^18^O	1614, 1395, 1224
	^12^C^16^O_2_	1650, 1436, 1229
	^13^C^16^O_2_	1609, 1399, 1227
	^13^C^18^O_2_	1594, 1382, 1217
ZrO_2_	^12^C^16^O	1626, 1429, 1331, 1221
	^13^C^18^O	1586, 1392, 1296, 1222
	^12^C^16^O_2_	1626, 1429, 1331, 1221
	^13^C^16^O_2_	1579, 1402, 1293, 1222
	^13^C^18^O_2_	1560, 1382, 1277, 1209

When ^13^C^18^O was adsorbed on ZrO_2_ the main carbonate bands appeared at 1586, 1392, 1296 and 1222 cm^−1^ (Table 3). The observed band frequencies are in between the carbonates formed from ^13^C^18^O_2_ and ^12^C^16^O_2_. They agree, however, with calculations for ^13^C^16^O vibrations, and are in reasonable agreement with carbonate frequencies detected upon adsorption of ^13^C^16^O_2_ (Table 3). The same trend had been observed before for alumina, which suggests the same mechanism of carbonate formation (scheme 1,). Since carbonate formation caused by water–gas-shift reaction should lead to formation of free ^13^C^16^O^18^O and thus to a mixture of ^13^C–^16^O and ^13^C–^18^O vibrations, which was however not observed, we suggested a preferred reaction route in which the ^13^C–^18^O bonds ends up in a configuration that is probably out of our IR range. For instance, ^13^C^18^O may react “oxygen down” with support oxygen vacancies or *cus*Al before it reacts with neighbouring surface OH groups to CO_2_, followed by immediate formation of carbonate species (Scheme 1).

## Conclusions

We have performed FTIR studies of the adsorption of unlabelled and isotopically-labelled CO and CO_2_ to examine the mechanism of carbonate formation on a series of Pd–alumina and Pd–zirconia catalysts. The catalysts were prepared from chlorine-free as well as from Cl-containing precursors and exhibited well-faceted (roughly) cubo-octahedral particles with mostly (111) and (100) surface facets and a low defect density. AES confirmed a concentration of about 2 wt% Cl originating from synthesis residues.

Carbonate formation is rather an activated process that occurs slowly below room temperature, but can be accelerated by the presence of Pd. Analysis of the carbonate vibrational frequencies as well as of the surface OH stretching range indicate carbonate formation via reaction of CO with surface OH (CO + OH → CO_2_ + ½ H_2_), with instantaneous CO_2_ reaction with the oxide support. The isotope studies further suggest an oxygen-down approach of CO, probably involving oxygen vacancies or defects of the alumina and zirconia support. The same reaction pathway is apparently active on the surface of both oxides. Carbonate formation via CO disproportionation could be excluded.

The effect of Cl contamination on carbonate formation was also studied. When surface OH groups were no longer available due to replacement by Cl (as detected by FTIR spectroscopy), carbonate formation was suppressed.

As a consequence, we propose that determining the concentration of carbonates formed from CO can be utilized to quantify the number of reactive surface defect sites on the oxide surface, thus being a sensitive and selective probe for defects. On γ-alumina approximately 1.4 × 10^12^ carbonate molecules were formed on 1 cm^2^ surface area or 1.4. × 10^− 2^ on 1 nm^2^ after pre-treatment at 573 K.
